# Molecular Epidemiology of Enterovirus in Children with Central Nervous System Infections

**DOI:** 10.3390/v13010100

**Published:** 2021-01-13

**Authors:** Lamprini Posnakoglou, Elizabeth-Barbara Tatsi, Panagiota Chatzichristou, Tania Siahanidou, Christina Kanaka-Gantenbein, Vasiliki Syriopoulou, Athanasios Michos

**Affiliations:** First Department of Pediatrics, Infectious Diseases and Chemotherapy Research Laboratory, Medical School, National and Kapodistrian University of Athens, “Aghia Sophia” Children’s Hospital, 11526 Athens, Greece; lina.posnakoglou@hotmail.com (L.P.); etatsi@med.uoa.gr (E.-B.T.); efthymia.chatzichristou@gmail.com (P.C.); siahan@med.uoa.gr (T.S.); ckanaka@med.uoa.gr (C.K.-G.); vsyriop@med.uoa.gr (V.S.)

**Keywords:** meningitis, encephalitis, FilmArray^®^, enterovirus, parechovirus, genotyping

## Abstract

Limited recent molecular epidemiology data are available for pediatric Central Nervous System (CNS) infections in Europe. The aim of this study was to investigate the molecular epidemiology of enterovirus (EV) involved in CNS infections in children. Cerebrospinal fluid (CSF) from children (0–16 years) with suspected meningitis–encephalitis (ME) who were hospitalized in the largest pediatric hospital of Greece from October 2017 to September 2020 was initially tested for 14 common pathogens using the multiplex PCR FilmArray^®^ ME Panel (FA-ME). CSF samples positive for EV, as well as pharyngeal swabs and stools of the same children, were further genotyped employing Sanger sequencing. Of the 330 children tested with FA-ME, 75 (22.7%) were positive for EV and 50 different CSF samples were available for genotyping. The median age of children with EV CNS infection was 2 months (IQR: 1–60) and 44/75 (58.7%) of them were male. There was a seasonal distribution of EV CNS infections, with most cases detected between June and September (38/75, 50.7%). EV genotyping was successfully processed in 84/104 samples: CSF (*n* = 45/50), pharyngeal swabs (*n* = 15/29) and stools (*n* = 24/25). Predominant EV genotypes were CV-B5 (16/45, 35.6%), E30 (10/45, 22.2%), E16 (6/45, 13.3%) and E11 (5/45, 11.1%). However, significant phylogenetic differences from previous described isolates were detected. No unusual neurologic manifestations were observed, and all children recovered without obvious acute sequelae. Specific EV circulating genotypes are causing a significant number of pediatric CNS infections. Phylogenetic analysis of these predominant genotypes found genetic differences from already described EV isolates.

## 1. Introduction

Enteroviruses (EVs) are members of the *Picornaviridae* family and consist of a non-enveloped positive single-stranded RNA [1]. The enterovirus genus contains four enterovirus species (A to D) and three rhinovirus species (A to C) and there is no zoonotic reservoir for EVs that infect humans [2,3].

Although EV infections are frequently asymptomatic, they can present with a variety of clinical manifestations comprising fever, exanthems, headache, respiratory illness, sore throat, myocarditis, vomiting, diarrhea and sepsis-like illness in neonates and infants [3]. Several members of EVs are neurotropic pathogens with a wide spectrum of clinical disorders ranging from aseptic meningitis to more severe encephalitis [3]. Although polio EVs have almost been eradicated due to systematic immunization, still circulating non-polio EVs are also associated with severe neurologic manifestations such as acute flaccid myelitis (AFM). Recent outbreaks associated with severe neurological complications and AFM have been reported in several countries from different continents including Europe and the USA, possibly attributed to EV-D68 and EV-A71 [4,5,6].

Single or multiplex PCR platforms including EV detection increased the diagnostic yield and our knowledge regarding enteroviral involvement in pediatric central nervous system (CNS) infections [7,8,9]. Further characterization of EV species with molecular sequence data, comparative genomics and phylogenetic analysis is important to identify the circulating and emerging EVs causing CNS infection or neurologic manifestations and elucidate epidemic outbreaks on time [10,11].

Limited data have been published regarding circulating EVs involved in pediatric CNS infection in Europe since 2017 [12]. Although in Greece, there is a reference center for enteroviruses based on the Hellenic Pasteur Institute, the last published data stop in 2015 [13,14]. The aim of this study was to investigate the molecular epidemiology of EV causing CNS infections in children during a 3-year period.

## 2. Materials and Methods

### 2.1. Study Cohort

This is a prospective cohort study which included children (0–16 years) who were admitted at Aghia Sophia Children’s Hospital, which is the largest tertiary pediatric Greek hospital, with possible clinical diagnosis of CNS infection from October 2017 to September 2020. The clinical suspicion was based on the age, on the clinical presentation with fever plus one or more of the following symptoms or signs: exanthem, convulsions, headache, vomiting, photophobia, altered mental status, nuchal rigidity, irritability or somnolence [15]. The setting of the study is a 750-bed tertiary pediatric hospital which serves almost 40% of the metropolitan Athens area and is a reference center for southern and central Greece. Infants and children who had a clinical diagnosis of CNS infection and whose cerebrospinal fluid (CSF) was obtained were included in the study.

Molecular detection methods as well as genotyping were performed in the Infectious Diseases Laboratory of the Choremeion Research facility.

The study protocol was approved by the scientific and bioethics committee of Aghia Sophia Children’s Hospital and informed consent was obtained by parents or legal guardians before children were included in the study.

### 2.2. Sample Collection and Molecular Screening Test

CSF was obtained from children with suspected meningitis–encephalitis (ME) by lumbar puncture to detect the causative CNS infection pathogen. CSF was tested with conventional microbiological procedures including CSF analysis (cells, protein, glucose) and bacterial culture and gram stain as well as BioFire FilmArray^®^ Meningitis/Encephalitis (FA-ME) Panel.

BioFire FilmArray^®^ Meningitis/Encephalitis (FA-ME) Panel (BioFire Diagnostics, Inc., Salt Lake City, UT, USA) is an automated multiplex PCR of 14 common pathogens for ME, including EVs. FA-ME was performed according to the manufacturer’s instructions in CSF samples. In children with an EV detection in CSF with the FilmArray^®^ ME panel, pharyngeal swabs and stool specimens were also collected for additional genotyping.

### 2.3. Isolation of Viral Genome and RT-PCR of VP1 Gene

Viral RNA genome was isolated from CSF, pharyngeal swabs and stools employing the MagNA Pure Compact Nucleic Acid Isolation Kit I (Roche Diagnostics, Basel, Switzerland) on the MagNA Pure Compact instrument or the QIAamp Viral RNA Mini Kit (Qiagen, Hilden, Germany) according to the manufacturer’s instructions. Only stool samples were prepared with 1.4 mL of Stool Transport and Recovery (S.T.A.R.) buffer (Roche Diagnostics, Basel, Switzerland) and 100 μL of chloroform followed by a centrifugation at 10,000 rpm for 5 min before the extraction method. cDNA synthesis was carried out using the Transcriptor First Strand cDNA Synthesis Kit (Roche Diagnostics, Basel, Switzerland) according to the manufacturer’s instructions and specific previously described primers [16]. Next, two rounds of PCR were performed according to Nix et al. with the addition of a ramping step in the first round PCR (60 °C (0.4 °C/s) for 45 s).

All primers that were used in this study were designed according to the genome of poliovirus type 1, Mahoney strain for EVs, as described by Nix et al., according to WHO guidelines [16].

### 2.4. Sanger Sequencing

Genotyping of EV strains was carried out by performing Sanger sequencing with the BigDye Terminator v3.1 Cycle Sequencing Kit on an Applied Biosystems 3500 Genetic Analyzer (Applied Biosystems, Waltham, MA, USA). The electrochromatographic data of sequencing were analyzed with BLAST (https://blast.ncbi.nlm.nih.gov/) to find the genotype of EVs and HPeVs.

### 2.5. Phylogenetic Analysis

Phylogenetic evolutionary analysis was performed using the MEGA X 10.2.0 software (Molecular Evolutionary Genetics Analysis; www.megasoftware.net) [17]. Global reference sequences of the *VP1* gene of E30, CV-B5 and E16 strains isolated from human were obtained from the genetic sequence database GenBank (https://www.ncbi.nlm.nih.gov/genbank/). The sequenced part of the *VP1* gene from the E30, CV-B5 and E16 strains of the present study and the corresponding reference sequences of *VP1* genes were multiple aligned using MUSCLE software (MUltiple Sequence Comparison by Log-Expectation). Phylogenetic trees were constructed using the neighbor-joining statistical method and bootstrap resampling with 1000 replicates. The used substitution model was the Maximum Composite Likelihood model. The strains Frater (AF081341), Faulkner (AF114383) and Harrington (AF295503) were used as outgroups for rooting the E30, CV-B5 and E16 trees, respectively.

### 2.6. Statistics Analysis

Statistical analysis was performed using the SPSS v.25 software (IBM Corp.). *p* value < 0.05 was considered statistically significant. The data are expressed as median and interquartile range (IQR) unless differently indicated. Comparison among groups was performed using the Kruskal–Wallis test with Bonferroni’s post-hoc analysis.

## 3. Results

**Figure 1:**
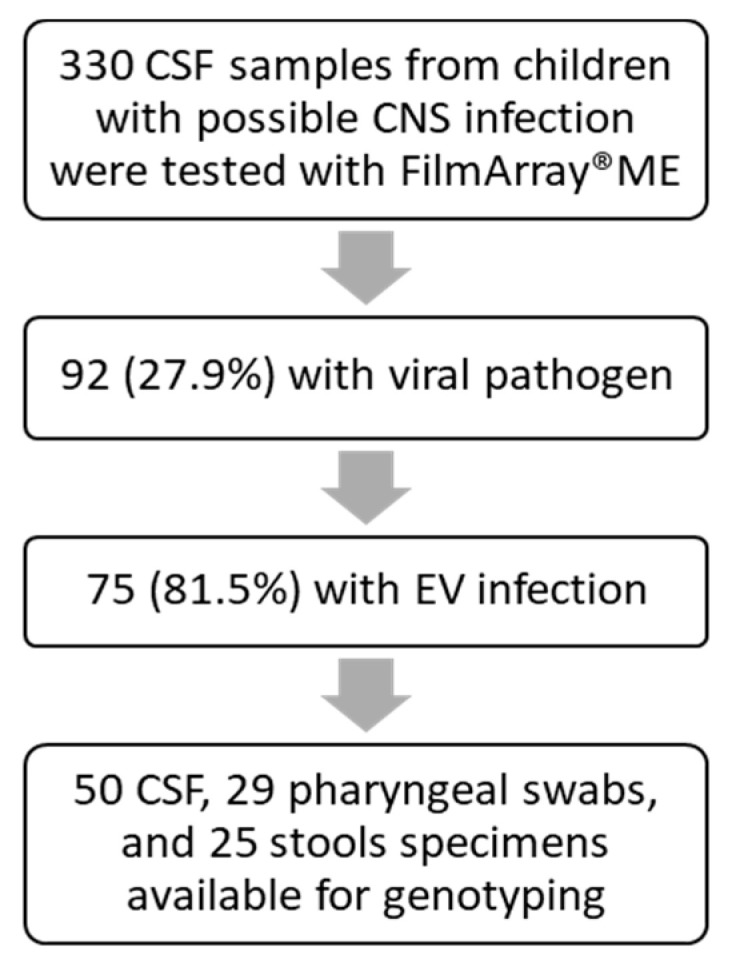
The workflow of available cerebrospinal fluid (CSF) samples from children with suspected meningitis/encephalitis that were included in this study.

During the 3-year study period, a total of 330 different CSF samples were tested with the multiplex PCR FilmArray^®^ ME panel and 13 (4%) bacterial and 92 (27.9%) viral pathogens were detected. Among CSF samples with a viral pathogen detection, EV was detected in 75/92 (81.5%) (Figure 1).

Except EVs, other viral pathogens were detected with FA-ME in 17 children: human herpesvirus 6 (HHV-6) in 11/92 (12%) and human parechovirus (HPeV) in 6/92 (6.5%). Most children with HHV-6 CNS infection (10/11, 90.9%) presented with fever and exanthem as prevailing clinical manifestations. All children with HPeV CNS infections were neonates and young infants who presented with acute febrile disease, 3/6 presented with sepsis-like illness, 3/6 with rash and 2/6 with seizures. CSF analysis of HPeV-positive neonates found absence of pleocytosis.

**Figure 2:**
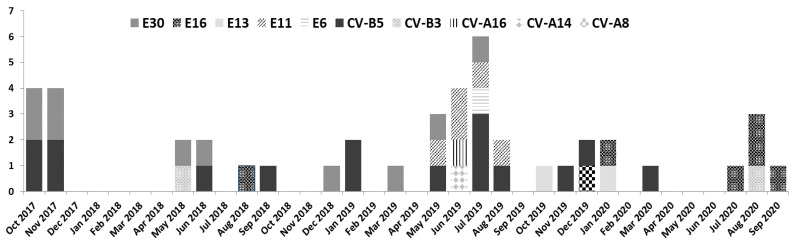
Seasonal and annual distribution of the 10 enterovirus (EV) genotypes detected in 45 children with EV CNS infection (October 2017–September 2020).

Seventy-five children with a median age of 2 months (IQR: 1–60) were infected with EV and 58.7% (44/75) of them were male. The annual distribution of EV cases among viral infections was 11/12 (91.6%) in 2017, 23/27 (85.1%) in 2018, 31/42 (73.8%) in 2019 and 10/11 (90.9%) in 2020. There was a seasonal distribution of EV CNS infections, with most cases detected in June 11/75 (14.6%), July 11/75 (14.6%), August 8/75 (10.6%) and September 8/75 (10.6%) (Figure 2).

The most common clinical symptoms in children with EV CNS infection were fever (72/75, 96%), headache (24/75, 32%), vomiting (18/75, 24%) and photophobia (13/75, 17%). The most common clinical signs were nuchal rigidity (20/75, 27%) and exanthem (7/75, 9%).

The laboratory analysis of CSF samples of children with EV CNS infection revealed (median, IQR): white blood cells (WBCs), 155 cells/mm3 (IQR: 30–342); neutrophils, 33% (IQR: 19.3–45.5); lymphocytes, 58% (IQR: 29–69); glucose, 48.5 mg/dl (IQR: 38–56); protein, 48.15 g/dl (IQR: 33–83). The median number of days in hospitalization was 4 days (IQR: 3–5) and the duration of their antibiotic treatment was 2 days (IQR: 1–4).

From 75 different CSF samples positive for EV, 50 had sufficient quantity available and pharyngeal swabs and stools were also collected for further genotyping (Table 1). EV genotyping was successfully performed in 84/104 different samples from 50 different EV-positive children and, more specifically, in 45/50 (90%) CSF samples, in 15/29 (52%) pharyngeal swabs and in 24/25 (96%) available stool specimens, indicating that CSF and stool samples are the most suitable biological samples for ME diagnosis. The EV genotypes detected in different available samples were identical in all cases.

Sanger sequencing revealed ten different genotypes circulating among 45 children with suspected ME (Table 1). The most prevalent genotypes were CV-B5: 16 (35.6%); E30: 10 (22.2%); E16: 6 (13.3%); E11: 5 (11.1%).

The annual distribution analysis of EV genotypes demonstrated a predominance of E30 (3/7, 42.9%) and CV-B5 (9/22, 40.9%) in 2018 and 2019, respectively, which were replaced by E16 (5/8, 62.5%) in 2020 (Figure 2).

Regarding the clinical presentation, meningitis was predominant in CV-B5 (12/16), CV-A8 (1/1), CV-A14 (1/1), CV-A16 (1/1), CV-B3 (2/2), E30 (9/10), E6 (1/1) and E13 (2/2) infections. Encephalitis was observed in E11 (3/5) and in CV-B5 (2/16) infections. In children with EV CNS infection and sepsis-like presentation, genotypes CV-B5 (2/16), E16 (2/6) and E30 (1/10) were detected.

Comparison of CSF WBCs among the four predominant EV genotypes (CV-B5, E30, E16 and E11) demonstrated than children with E11 infection had fewer CSF WBCs (median value 3, IQR: 0–46 cell/mm^3^) and E30 the highest (median value 296, IQR: 82.75–471.25 cell/mm^3^) (*p*-value: 0.008).

Most children older than 1 month were infected by CV-B5 (14/16, 87.5%) and E30 (9/10, 90%), while children <1 month were infected by E11 (3/5, 60%) and E16 (4/6, 66.6%) (Table 1).

The most severe clinical presentation was in an 18-month-old boy with CV-A8 infection, who was presented to the Emergency Department of the hospital with fever, status epilepticus and coma. During the study period, no unusual neurologic manifestations were observed and all children with EV CNS infection recovered without obvious sequelae.

**Figure 3:**
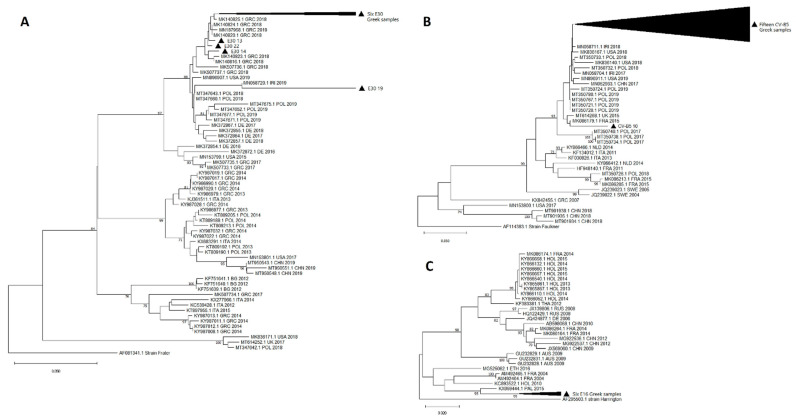
Phylogenetic trees of E30 (**A**), CV-B5 (**B**) and E16 (**C**) strains of this study with other global reference strains based on a part of 308, 274 and 295nt of the *VP1* gene, respectively. Greek strains of this study are indicated with a filled black triangle. Outgroups are indicated by name and accession number from GenBank. The rest of the reference strains are labeled with the accession number, 2 or 3 letters of the country of origin and the year of isolation. The neighbor-joining method and bootstrap test of 1000 replicates were used to infer the phylogenetic trees. Only percentage values of replicate trees above 70% are shown next to branches. Notes: GRC = Greece; HOL = Holland; POL = Poland; DE = Germany; FRA = France; ITA = Italy; UK = United Kingdom; BG = Bulgaria; AUS = Australia; RUS = Russia; CHN = China; THA = Thailand; PAL = Palestine; IRI = Iran; ETH = Ethiopia.

Phylogenetic analysis was performed for the predominant strains CV-B5 (*n* = 16), E30 (*n* = 10) and E16 (*n* = 6) to clarify the genetic relationships and evolutionary history among them and other strains circulating globally during the same period (Figure 3).

All E30 Greek samples from the present study fell into the same subcluster of other Greek and European strains (Germany and Poland) isolated within 2017–2019, which shared a nucleotide similarity of 88%. However, the sample E30 (case 19) had a similarity of 61% with the unpublished Iran strain (MN058720) isolated from stool in 2019.

CV-B5 Greek samples were also clustered with other European (France, United Kingdom and Poland) and non-European (USA, Iran and China) strains isolated during the 2015–2019 period with a genetic similarity of 93%. All 16 EV CV-B5 strains seem to be mainly related to Polish strains from 2019, despite a similarity of <70%. Isolate CV-B5 (case 10) was distinguished from the other 15 ones of our study since it was classified closer to the French strain (MK086179) isolated in 2015 from a 40-year-old male with meningitis. This was an isolate from a 3-month-old infant who presented with meningoencephalitis, and enterovirus and pneumococcus were detected simultaneously in CSF by FilmArray^®^ ME.

There have not been previously reported E16 Greek strains in the GenBank database, and therefore, only other globally reported strains were included in the phylogenetic analysis. Greek E16 samples of this study appear to have similar evolutionary distance from the common ancestor and a nucleotide similarity of 95% with the Palestine strain (KX059444) from a CSF sample isolated in 2015. A nucleotide divergence of approximately 50% was noticed between Greek E16 samples and other European (France and Holland) and global (Asia, Africa and Australia) strains isolated up until 2016.

## 4. Discussion

In the present study, we describe the molecular epidemiology of EVs in children with CNS infections from 2017 to 2020. During the study period, EVs were the main viral pathogens involved in aseptic meningitis/encephalitis cases (81.5%).

Initial identification of EVs was made through a syndromic multiplex CSF PCR platform, which includes EV detection and has been shown to duplicate the diagnostic yield in CNS infections compared to the control group [8]. In a previously published study from our group, this was found to be particularly important for discontinuing unnecessary antibiotics and antivirals in neonates and older children with viral ME [8].

The reverse-transcriptase polymerase chain reaction (RT-PCR), either as a single PCR or in multiplex PCR platforms, is now the “gold standard” for diagnosing EV infections due to its advantages of a fast turn-around time and high sensitivity over virus isolation [18]. However, different protocols have been developed for the detection of EVs, and some commercially available platforms lack sensitivity, especially for the detection of low numbers of EV copies [18]. Because CSF contains low EV copies, it is suggested that additional testing from respiratory, blood or fecal samples should be performed for genotyping [19]. In the present study, except CSF, EV detection in stool was much higher compared to pharyngeal samples, and in all cases, the EV genotype was identical among the three biological samples. This finding could be useful for pediatricians as CSF quantity is usually limited for PCR testing, especially in neonates and young infants.

Even with sensitive single or multiplex PCR protocols, a percentage of CSF EV infections could be missing. Inclusion of virus isolation from CSF samples using a few permissible cell lines (especially neuro-origin cell lines) could help in the discovery of potential novel neuro-invasive EV genotypes [20,21].

EVs demonstrate a marked seasonality in temperate climates, with a typical EV high season occurring during June–November [22,23,24]. The major EV genotypes identified in our study were Coxsackievirus B5, Echovirus 30 and Echovirus 16. In Greece, EV meningitis outbreaks or dominant EV genotypes were identified in 2001 (Echovirus 6), in 2005 (Echovirus 15), in 2006 (Echovirus 6) and in 2007 (Echovirus 4) [23,25,26,27,28,29]. Smaller outbreaks in neonatal units were reported in 2011 (Echovirus 6) and in 2013 (Echovirus 30) [30,31].

Limited data are available regarding circulating EV genotypes which are associated with pediatric CNS infections in Europe during this period. In a Polish study, from January 2015 to December 2019, in 188 children, 19 different enterovirus types were identified, and Coxsackievirus B5 (32%), echovirus 30 (20%), and echovirus 6 (14%) were the three most common types [12]. Coxsackievirus B5 is reported as a major cause of viral meningitis in Ireland, Spain, and China [32,33,34].

Enteroviral infections seem to have tissue tropism and different genotypes are associated with various clinical manifestations [35]. During the study period, there was not any identification of EV-A71 and EV-D68, which are associated with severe neurological manifestations such as acute flaccid myelitis [36,37]. The characterization of emerging EV subtypes which are associated with unusual clinical presentations could help elucidate epidemic outbreaks or document new genetic variants with high virulence.

Echovirus 30 was the predominant EV genotype in our area in 2018. In 2020, due to the COVID-19 pandemic and the social distancing measures and school closures, there were fewer cases of aseptic meningitis, while genotype Ε16 predominance was detected. Echovirus meningitis outbreaks have been reported to occur worldwide every 3 to 5 years [38,39,40]. The most recent EV meningitis epidemics in Europe occurred in Germany (Echovirus 30), in northern European countries in 2013–2014 (Echovirus 4) and in 2018 (Echovirus 30) [11,41,42]. However, sequencing data from other studies have suggested that there are genetic exchanges between different isolates, and multiple genotypes could circulate simultaneously in the same outbreak [43].

CV-B5 and echoviruses (6, 11, 30) are commonly associated with both encephalitis and aseptic meningitis, as it was also identified in our population [44]. Likewise, CV (A5, A7, A16, B2, B3, B4) and echoviruses (14, 16, 25, 31) are all implicated in cases of meningitis [44,45]. However, in our population, E16 was not only associated with meningitis but also with two cases of neonatal sepsis-like disease.

In the present study, we report, for first time, a child with severe CV-A8 infection who presented with fever, status epilepticus and coma. CV-A8 infections were previously associated with hand, foot and mouth disease (HFMD), herpangina (HA) or mild neurological manifestations [46,47].

In this study, a preliminary phylogenetic analysis of E30, CV-B5 and E16 strains was performed and indicated a possible correlation with other strains mainly circulating in European countries such as Poland (E30, CV-B5), France (CV-B5) and Germany (E30) as well as in non-European countries such as Iran (E30, CV-B5) and Palestine (E16), which could be attributed to the population movement. To estimate the genetic evolution of these pathogens, whole-genome sequencing followed by a phylogenomic analysis is required.

Genetic heterogeneity was noticed even among the closest strains. Interestingly, in the E30 phylogenetic tree, genetic divergences occurred even between Greek strains of this study and reported Greek strains of the same period. This phenomenon may reflect the evolving dynamic of this pathogen.

The Greek E16 strains of our study may have undergone nucleotide variants which classify them closer to the ancestor strain, while simultaneously significantly differentiating them from circulating strains in other countries. The highly genetic homology between the predominant Greek E16 isolates in 2020 may indicate a small outbreak of the specific E16 strain in Greece.

Although all children with EV CNS infection in our study recovered without obvious acute sequelae, there was not any neurodevelopmental follow-up. There is lack of large prospective studies regarding the long-term complications in children with EV ME, especially in the neonatal period. However, there are case-series indicating that neurological sequelae could occur such as neurodevelopmental delay, impaired verbal function, seizures and epilepsy [48,49,50].

## 5. Conclusions

In conclusion, fast and accurate molecular identification of enteroviruses is essential for the accurate diagnosis of viral pediatric ME, as enteroviruses are the main viral pathogen involved. Further genetic characterization of circulating EVs causing CNS infection or neurologic manifestations could elucidate association of specific genotypes with specific clinical manifestations and allow to recognize epidemic outbreaks on time. For these reasons, continuous epidemiological surveillance for emerging enteroviral genotypes is required and large-scale epidemiological studies should be conducted.

## Figures and Tables

**Table 1:** Enteroviral (EV) genotype detection and age distribution in 84/104 different types of samples, from children with EV CNS infection (*n* = 50). Ten different EV genotypes were detected during the study period (October 2017–September 2020). m: months; y: years.

EV Genotypes	No of Children *n* (%)	Age			CSF	Pharyngeal Swab	Stool
		<1 m *n*	1 m–1 y *n*	>1 y *n*			
CV-A8	1 (2.2)			1	1	1	1
CV-A14	1 (2.2)	1			1	1	1
CV-A16	1 (2.2)		1		1	0	1
CV-B3	2 (4.4)	1		1	2	0	0
CV-B5	16 (35.6)	2	8	6	16	4	6
E6	1 (2.2)		1		1	0	1
E11	5 (11.1)	3	2		5	4	5
E13	2 (4.4)	1	1		2	0	2
E16	6 (13.3)	4		2	6	2	4
E30	10 (22.2)	1	3	6	10	3	3
Samples Genotyped	45/50	13/45	16/45	16/45	45/50	15/29	24/25

## Data Availability

Study data are available upon request.
